# Anti-nociceptive and desensitizing effects of olvanil on capsaicin-induced thermal hyperalgesia in the rat

**DOI:** 10.1186/s40360-016-0074-9

**Published:** 2016-07-21

**Authors:** Mohammad Alsalem, Paul Millns, Ahmad Altarifi, Khalid El-Salem, Victoria Chapman, David A. Kendall

**Affiliations:** Department of Anatomy and Histology, Faculty of Medicine, The University of Jordan, Amman, 11942 Jordan; School of Life Sciences, University of Nottingham, Queen’s Medical Centre, Nottingham, NG7 2UH UK; Faculty of Medicine, Jordan University of Science and Technology, Irbid, 22110 Jordan; Arthritis Research UK Pain Centre, Nottingham, UK

**Keywords:** Transient receptor potential vanilloid type 1, Cannabinoid receptor 1, Pungency and pain

## Abstract

**Background:**

Olvanil (NE 19550) is a non-pungent synthetic analogue of capsaicin, the natural pungent ingredient of capsicum which activates the transient receptor potential vanilloid type-1 (TRPV1) channel and was developed as a potential analgesic compound. Olvanil has potent anti-hyperalgesic effects in several experimental models of chronic pain. Here we report the inhibitory effects of olvanil on nociceptive processing using cultured dorsal root ganglion (DRG) neurons and compare the effects of capsaicin and olvanil on thermal nociceptive processing in vivo; potential contributions of the cannabinoid CB_1_ receptor to olvanil’s anti-hyperalgesic effects were also investigated.

**Methods:**

A hot plate analgesia meter was used to evaluate the anti-nociceptive effects of olvanil on capsaicin-induced thermal hyperalgesia and the role played by CB_1_ receptors in mediating these effects. Single cell calcium imaging studies of DRG neurons were employed to determine the desensitizing effects of olvanil on capsaicin-evoked calcium responses. Statistical analysis used Student’s t test or one way ANOVA followed by Dunnett’s *post-hoc* test as appropriate.

**Results:**

Both olvanil (100 nM) and capsaicin (100 nM) produced significant increases in intracellular calcium concentrations [Ca^2+^]_i_ in cultured DRG neurons. Olvanil was able to desensitise TRPV1 responses to further capsaicin exposure more effectively than capsaicin. Intraplantar injection of capsaicin (0.1, 0.3 and 1 μg) produced a robust TRPV1-dependant thermal hyperalgesia in rats, whilst olvanil (0.1, 0.3 and 1 μg) produced no hyperalgesia, emphasizing its lack of pungency. The highest dose of olvanil significantly reduced the hyperalgesic effects of capsaicin in vivo. Intraplantar injection of the selective cannabinoid CB_1_ receptor antagonist rimonabant (1 μg) altered neither capsaicin-induced thermal hyperalgesia nor the desensitizing properties of olvanil, indicating a lack of involvement of CB_1_ receptors.

**Conclusions:**

Olvanil is effective in reducing capsaicin-induced thermal hyperalgesia, probably via directly desensitizing TRPV1 channels in a CB_1_ receptor-independent fashion. The results presented clearly support the potential for olvanil in the development of new topical analgesic preparations for treating chronic pain conditions while avoiding the unwanted side effects of capsaicin treatments.

## Background

TRPV1 (transient receptor potential vanilloid type 1) is a chemically-gated non-specific cation channel with high calcium permeability [1], which integrates a range of painful stimuli including noxious heat (>43 ° C) [2] and low pH [3] and which responds to pungent compounds, such as capsaicin [2, 4]. TRPV1 is widely distributed throughout the central [5] and peripheral nervous systems [6]. It is well established that TRPV1 is a key molecule involved in the progress of many chronic pain conditions such as those associated with inflammation, cancer and neuropathy [7–10]. As a consequence, TRPV1 is viewed as a prime target for pharmacological intervention to control pain. Although the development of TRPV1 antagonists has been a logical step forward for developing new analgesics, their clinical benefit is limited by unwanted side effects including effects on core body temperature [11–13].

The increase in intracellular calcium concentration ([Ca^2+^]_i_) mediated by TRPV1 is essential to induce neurotransmitter release from the primary afferent fibres. However, elevation of [Ca^2+^]_i_ through TRPV1 activation leads to desensitization of the channel, probably through calcium-dependent mechanisms including Ca^2+^/calmodulin and the Ca^2+^-sensitive phosphatase calcineurin [14, 15]. Desensitization of TRPV1, which occurs after the calcium entry, provides a feedback mechanism to regulate calcium homeostasis and is the basis of the use of TRPV1 agonists as analgesics as an alternative approach to TRPV1 antagonism. Indeed, topical preparations containing low concentrations of capsaicin have long been used as over-the-counter remedies for pain.

Clinical trials have demonstrated that capsaicin is effective in alleviating pain associated with osteoarthritis [16], and a synthetic, orally active isomer of capsaicin, civamide, was reported to significantly reduce cluster headache [17] and migraine headache [18]. High-concentration topical capsaicin (8 % patch; Qutenza®) is licensed in the EU to treat neuropathic pain in non-diabetic patients, and in the US to treat peripheral herpetic neuralgia. Treatment can give rise to a long-lasting effect, which has been termed ‘defunctionalisation’, probably owing to a number of different effects that, together, overwhelm neuronal homeostasis, leading to reversible degeneration of nerve terminals [19]. However, the use of TRPV1 agonists like capsaicin is hindered by the initial sharp and burning sensation, referred to as pungency which requires patches to be available for use only by specialist healthcare workers and needs pre-treatment with local anesthetic.

Development of non-pungent capsaicin analogues such as olvanil, palvanil and arvanil with retention of analgesic properties could be of significant clinical value. Previous studies explored differences between pungent and non-pungent TRPV1 agonists, and proposed various hypotheses to explain the translation of these differences in in vivo pungency. Interestingly, none of the previous reports evaluated the effects of the non-pungent TRPV1 agonist olvanil on the capsaicin-evoked calcium responses in isolated DRG neurons, which may have an important physiological relevance in particular to TRPV1 desensitization and the subsequent neurotransmitter release. Furthermore, the contribution of CB_1_ cannabinoid receptors to the analgesic effects of olvanil in vivo remains unclear, as the previous reports were focused solely on in vitro studies using cell lines. The aim of the current study was to fill in these gaps and test the anti-hyperalgesic effects of olvanil and to further study the potential role for cannabinoid receptors in mediating these analgesic effects. To address the mechanism of the desensitizing effects of olvanil on the capsaicin-evoked hyperalgesia, calcium responses in isolated DRG neurons were also evaluated.

## Methods

### Animals

Calcium imaging experiments used adult male Sprague Dawley rats (180–200 g, Charles River UK). Rats were group housed in the Biomedical Services Unit (University of Nottingham) in a temperature controlled environment 22 ± 1 °C at 12 h: 12 h light: dark cycle. Behavioural experiments used adult male albino rats (180–200 g, The University of Jordan laboratories). Procedures were approved by the University of Nottingham Ethical Review Committee and by the scientific research committee at the University of Jordan. Experiments were carried out in accordance with the Animal (Scientific Procedure) Act 1986 and International Association for the Study of Pain guidelines.

### Calcium imaging of dorsal root ganglion neurons

A total of 16 rats, killed by CO_2_ overdose, were used for the calcium imaging studies. Spinal columns were removed and dorsal root ganglia (DRGs) neurons were collected [20]; cell preparation and culture were as described previously [21, 22].

Cells were washed three times with calcium imaging buffer (NaCl, 145 mM; KCl, 5 mM; CaCl_2_, 2 mM; MgSO_4_.7H_2_O, 1 mM; HEPES, 10 mM; glucose, 10 mM; pH, 7.4). Then cells were loaded with 5 μl of Fura2-AM in 895 μl of calcium buffer with 100 μl of fetal calf serum (FCS) and incubated for 30 min in the dark. Cells were washed with calcium buffer and left for at least 15 min prior to imaging. [Ca^2+^]_i_ was measured as the ratio of peak fluorescence emission intensities (measured at 500 nm) at excitation wavelengths of 340 nm and 380 nm, respectively, using an Andor IQ imaging system and expressed as changes in relative fluorescence units (ΔRU). Coverslips were fixed to a Perspex chamber using vacuum grease, and the DRG neurons were suprafused with calcium buffer at a rate of 2 ml/min. All drugs were applied by suprafusion in calcium buffer.

### Effects of olvanil on capsaicin-evoked calcium responses

Experiments were designed so that DRG cells were not exposed to capsaicin more than three times. A standard protocol was followed in which DRG cells were exposed to capsaicin (100 nM) for 1 min followed by a 45 min wash-out period. Olvanil (100 nM) was then suprafused for 1 min followed by another 45 min of wash-out. Finally, cells were re-exposed to capsaicin (100 nM for 1 min) followed by 45 min of wash-out. This protocol was followed based on the observation that the majority of capsaicin-evoked calcium responses returned to baseline values after a 45 min wash-out period, as previously reported [22, 23].

To determine whether TRPV1 desensitization could have been a confounding factor, parallel control experiments were conducted in which DRG cells were challenged three times with capsaicin, using the same timing schedule as described above (capsaicin exposure separated by a 45 min wash-out period). A depolarizing concentration of KCl (60 mM) was applied at the end of each experiment to confirm neuronal responsiveness. KCl responding cells that had a peak 340:380 nm ratio of more than 0.10 and were at least 0.2 fluorescence units above the baseline were included in the analysis. Peak ratios were calculated by subtracting the baseline ratio from the ratio obtained during drug suprafusion (ΔRU; ratio units difference). Results are expressed as a percentage of the peak response evoked by the first capsaicin application and presented as mean ± SEM. For each of the drug application protocols performed for calcium imaging, DRG cells were collected from three to five rats, and at least one coverslip of DRG cells from each rat was used for each experiment.

### Hot plate testing

Rats were placed individually on a hot plate analgesia meter (Columbus instruments, USA) maintained at a constant temperature of 55 ± 0.1 °C after observing them for 5 min in the cage [24]. The paw withdrawal latency (PWL) was recorded as the time taken to exhibit distinct pain behaviour either by hindpaw licking or hindpaw flicks (whichever occurred first). Rats that did not respond within 30 s were removed from the hot plate to prevent tissue damage [25].

### Capsaicin-induced thermal hyperalgesia

After determining the baseline latencies, rats received intraplantar injections of capsaicin (0.1, 0.3 and 1 μg in 100 μL of PBS or vehicle) under brief ether anesthesia. Thermal paw withdrawal latencies (PWL) were then determined at 10, 30 and 100 min after capsaicin injection. Data are presented as percentages of the baseline PWL (% PWL = Observed PWL / Baseline PWL × 100).

### Pharmacological treatments

For the assessments of the effects of different drugs on the capsaicin-induced thermal hyperalgesia, olvanil (0.1, 0.3 and 1 μg), 1 μg capsazepine, 1 μg rimonabant (a selective cannabinoid CB_1_ receptor antagonist) or olvanil (0.3 μg) plus rimonabant (1 μg) were injected 15 min prior to capsaicin; all drugs were dissolved in 50 μl of PBS, except capsaicin which was dissolved in 100 μl of PBS as described above. The maximum hyperalgesic effect of capsaicin was observed 10 min post-injection; therefore, all nociceptive testing was done 10 min following capsaicin administration.

### Drugs

Capsaicin, capsazepine, olvanil and rimonabant were purchased from Tocris Bioscience (UK). Drugs were initially dissolved in ethanol (100 %) to form a stock solution (capsaicin 1 mM, capsazepine 10 mM, olvanil 10 mM and rimonabant 10 mM), then diluted in phosphate-buffered saline (PBS). Final ethanol concentrations in the solutions used in the present study did not exceed 0.1 %.

### Data analysis

Calcium imaging data are presented as a means ± SEM of percentage of the peak responses evoked by the first capsaicin application, and statistical analysis (Prism 5, GraphPad Software Inc., La Jolla, CA, USA) used Student’s t-tests. For the studies measuring capsaicin-induced thermal hyperalgesia data are presented as means ± SEM of percentages of the baseline PWL (% PWL = Observed PWL/Baseline PWL× 100), and statistical analysis (Prism 5, GraphPad) used one way ANOVA test followed by Dunnett’s *post-hoc* test as appropriate.

## Results

### Capsaicin-induced thermal hyperalgesia

**Fig. 1:**
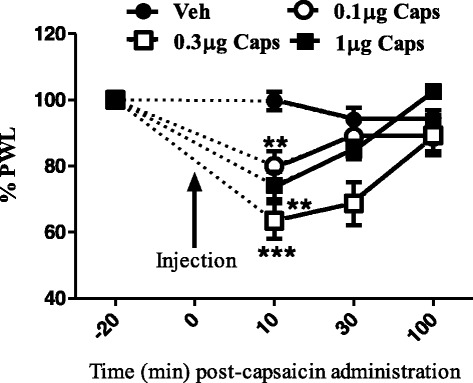
Effects of intraplantar injection of different doses of capsaicin on thermal paw withdrawal latency. Data are expressed as mean ± SEM of % PWL and analyzed using one way ANOVA test followed by Dunnett’s *post-hoc*, all doses of capsaicin were compared to vehicle at 10 min after injection (**P < 0.01, ***P < 0.001, *n* = 7 rats per group)

In the in vivo behavioral studies, intra-plantar injection of capsaicin produced a dose- and time-dependent hyperalgesia (Fig. 1). At all doses tested, the peak hyperalgesic effect was at 10 min post-injection, with the paw withdrawal latencies returning to baseline levels by 100 min post-injection.

**Fig. 2:**
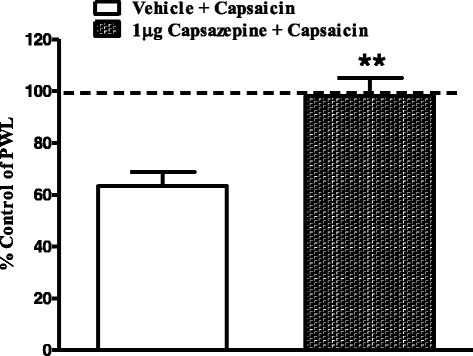
Effects of intraplantar injection of capsazepine (1 μg) on capsaicin-induced thermal hyperalgesia. Rats received intraplantar injections of capsazepine, or vehicle 15 min prior to capsaicin. Data are expressed as mean ± SEM of %PWL and analyzed using Student’s t test, (**P < 0.005, *n* = 7 rats per group)

To test whether the hyperalgesic effects of capsaicin were TRPV1 receptor-mediated, the TRPV1 receptor antagonist capsazepine (1 μg) was injected into the ipsilateral hindpaw of the rat 15 min prior to capsaicin. Capsazepine significantly attenuated capsaicin-induced thermal hyperalgesia (Fig. 2).

**Fig. 3:**
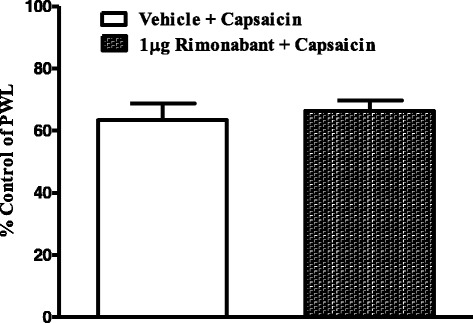
Effects of intraplantar injection of rimonabant (1 μg) on capsaicin-induced thermal hyperalgesia. Rats received intraplantar injections of rimonabant or vehicle 15 min prior to capsaicin. Data are expressed as mean ± SEM of %PWL and analyzed using Student’s t test (*n* = 7 rats per group)

To test whether an endogenous cannabinoid tone modulates the capsaicin-evoked thermal hyperalgesia, the selective CB_1_ antagonist rimonabant (1 μg) was injected into the ipsilateral hindpaw of the rat 15 min prior to capsaicin. Unlike capsazepine, rimonabant had no significant effect on capsaicin-induced thermal hyperalgesia (Fig. 3).

**Fig. 4:**
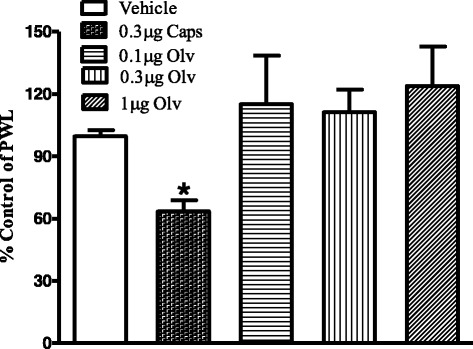
Effects of intraplantar injection of capsaicin (0.3 μg), olvanil (0.1, 0.3 and 1 μg), or vehicle on paw withdrawal latency. Data are expressed as mean ± SEM of % PWL and analyzed using one way ANOVA test followed by Dunnett’s *post-hoc*, all treatments were compared to vehicle at 10 min after injection (*P < 0.05, *n* = 7 rats per group)

**Fig. 5:**
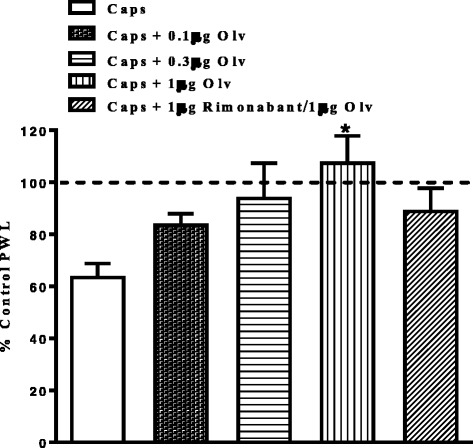
Effects of intraplantar injection of various doses of olvanil and rimonabant (1 μg) on capsaicin-induced thermal hyperalgesia. Rats received intraplantar injection of olvanil (Olv; 0.1, 0.3 and 1 μg) or a combination of 0.3 μg olvanil/1 μg rimonabant or vehicle 15 min prior to capsaicin (Caps). Data are expressed as mean ± SEM of % PWL and analyzed using one way ANOVA test followed by Dunnett’s *post-hoc*, different treatments were compared to vehicle at 10 min after injection (*P < 0.05, *n* = 7 rats per group)

In contrast to capsaicin, intra-plantar injection of olvanil (0.1, 0.3 and 1 μg) did not alter thermal nociceptive thresholds, confirming its lack of pungency (Fig. 4). Interestingly, intra-plantar injection of olvanil (1 μg) significantly attenuated capsaicin-evoked thermal hyperalgesia (Fig. 5). In order to evaluate the possible role of CB_1_ receptors in these inhibitory effects of olvanil, 1 μg rimonabant was co-administered with olvanil, The CB_1_ antagonist did not significantly alter the inhibitory effect of olvanil on capsaicin-evoked thermal hyperalgesia (Fig. 5).

### Olvanil attenuates capsaicin-evoked calcium responses

**Fig. 6:**
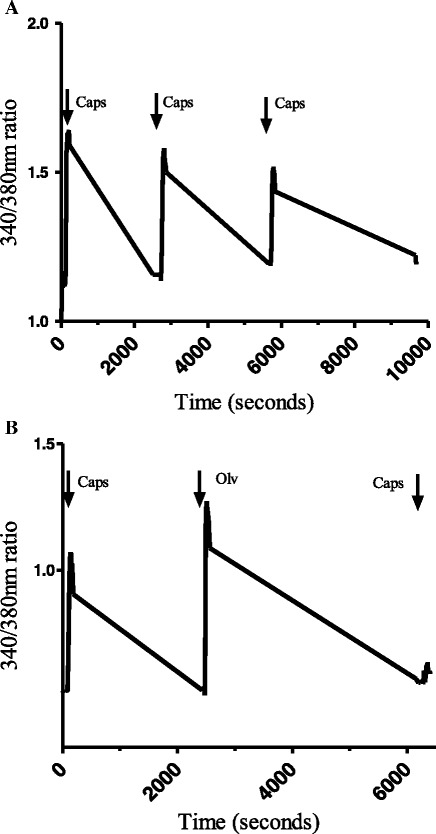
Representative traces illustrating changes in 340/380 nm ratios in DRG cells responding to capsaicin and olvanil. **a** The cell was suprafused with capsaicin (100 nM for 1 min) three times separated by 45 min of wash-out with calcium buffer. **b** The cell was exposed to capsaicin (100 nM) for 1 min, 45 min later, olvanil (100 nM) was applied for 1 min followed by another 45 min of wash-out. Finally, the cell was exposed again to capsaicin (100 nM for 1 min) followed by 45 min of wash-out

**Fig. 7:**
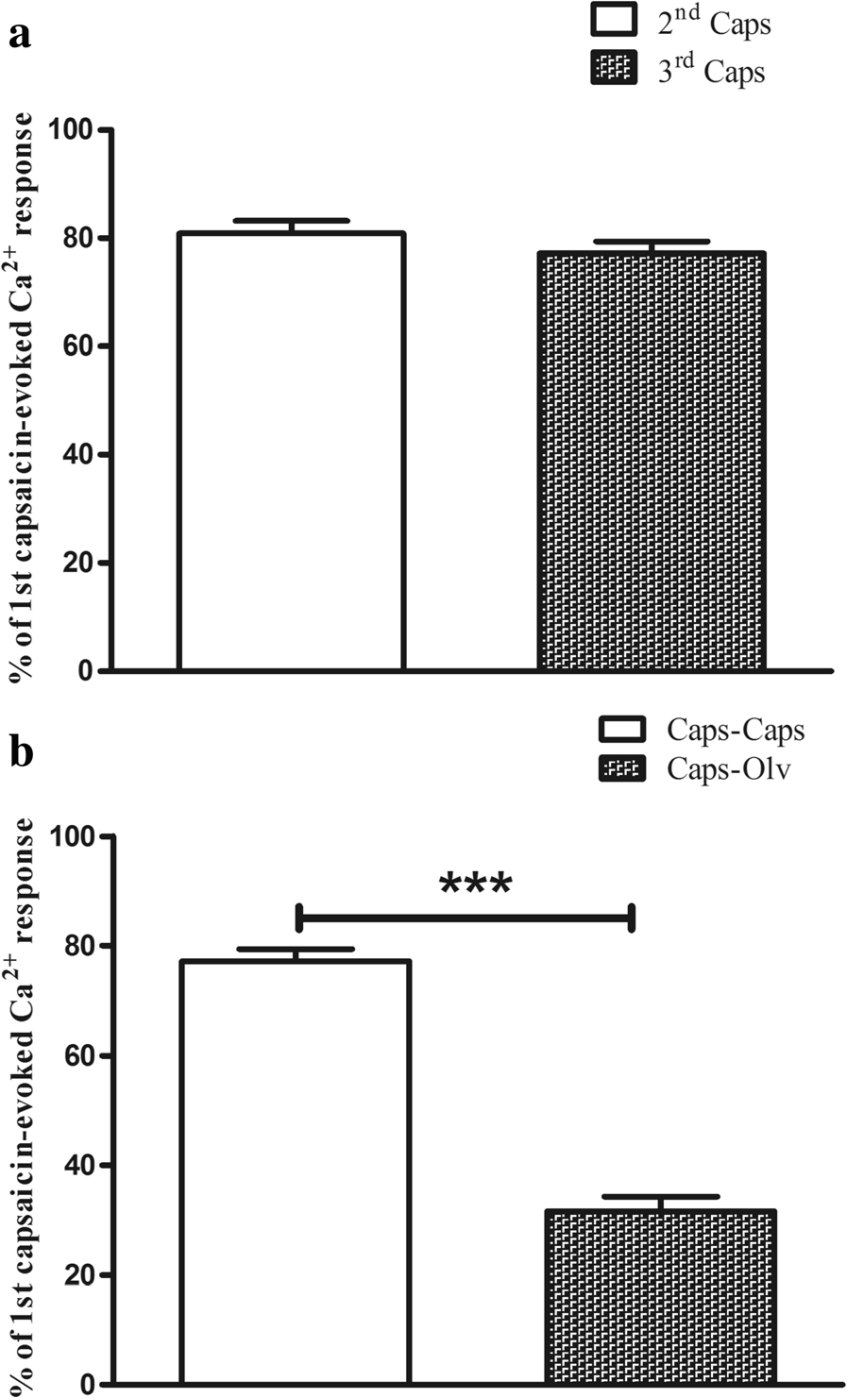
Capsaicin-evoked calcium responses in the whole population of DRG cells in the presence or absence of olvanil. Results are expressed as a percentage of the peak responses evoked by the first capsaicin application and are presented as means ± SEM. **a**, TRPV1-mediated calcium responses due to repeated exposure to capsaicin 100 nM. **b** olvanil significantly decreased the capsaicin-evoked calcium responses compared to an identical protocol of repeated capsaicin exposure in the presence of vehicle (***P < 0.001, Student’s t test)

Capsaicin produced a robust increase in [Ca^2+^]_i_ compared to basal (0.625 ± 0.03 ΔRU, n = 76 neurons, Fig. 6). To ensure that studies were not compromised by desensitization of TRPV1, effects of repeated application of capsaicin were studied. A second capsaicin challenge in the presence of vehicle (0.01 % ethanol in PBS) evoked a calcium response of 80.8 ± 2.3 % of the preceding capsaicin-evoked response. A third capsaicin challenge evoked a calcium response of 77.2 ± 2.2 % of the first capsaicin-evoked response. Statistical analysis revealed no significant differences between the second and third exposures to capsaicin (Fig. 7).

Olvanil produced a robust increase in [Ca^2+^]_i_ over basal (0.566 ± 0.04 ΔRU, *n* = 128 neurons). Pre-exposing the cells to the potent selective TRPV1 antagonist 5′-iodoresiniferatoxin (1 μM) completely blocked both capsaicin and olvanil-evoked calcium responses (data not shown). Statistical analysis revealed no significant differences between capsaicin-evoked calcium responses (0.625 ± 0.03 ΔRU, *n* = 76 neurons) and olvanil-evoked calcium responses (0.566 ± 0.04 ΔRU, *n* = 128 neurons, *P* value = 0.26) at the same concentration (100 nM); however, capsaicin-evoked calcium responses were significantly inhibited in cells pre-treated with olvanil (31.6 ± 2.6 % of the first capsaicin-evoked calcium response) compared to vehicle pre-treatment (77.2 ± 2.2 %, Figs. 6 and 7).

**Fig. 8:**
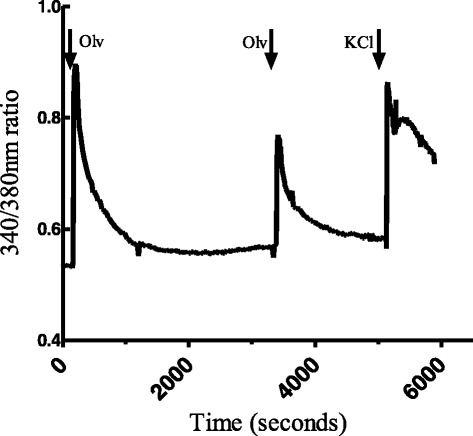
Representative traces illustrating changes in 340/380 nm ratios in DRG cells responding to olvanil. The cell was suprafused with olvanil (100 nM for 1 min) two times separated by 45 min of wash-out with calcium buffer. Finally, the cell was exposed to KCl (60 mM for 1 min) followed by 45 min of wash-out

In order to investigate whether the ability of olvanil to reduce the subsequent effects of capsaicin was simply due to a persistent desensitization of TRPV1, we studied the effect of repeated applications of olvanil on the DRG neurons. A second application of olvanil in the presence of vehicle (0.01 % ethanol) evoked a calcium response of 60.2 ± 1.6 % of the preceding olvanil-evoked calcium response (Fig. 8).

## Discussion

In the present study we investigated the anti-hyperalgesic effects of olvanil in a model of thermal hyperalgesia which employed capsaicin as an agent that directly activates TRPV1 on primary sensory neurons. In parallel, by means of single cell ratiometric calcium imaging experiments, we investigated TRPV1 receptor desensitization following exposure to olvanil.

In agreement with previous reports [26–28], intraplantar injection of capsaicin produced a robust thermal hyperalgesia in a TRPV1-dependent manner. After confirming the lack of pungency of olvanil using the same model, we showed that olvanil inhibited the hyperalgesic effects of intraplantar injection of capsaicin. Based on previous reports suggesting that olvanil may behave as a CB_1_ receptor agonist [29] we explored the mechanisms that underlie the anti-hyperalgesic effects of olvanil by evaluating the potential role of the CB_1_ receptor in mediating these effects of olvanil. The hypothesis that other receptors activated by olvanil, but not capsaicin, such as the cannabinoid CB_1_ [29] may account for the differences between these agonists in terms of pungency and could conceivably explain the anti-hyperalgesic effects of olvanil. However, the CB_1_ antagonist rimonabant did not alter the analgesic effects of olvanil, excluding a role for CB_1_ in mediating these effects in vivo. This is in spite of previous in vitro studies using cell lines that reported olvanil binding to CB_1_ receptors and inhibition of adenylyl cyclase leading to reduction of cAMP levels in N18TG2 neuroblastoma cells [30]. In addition, olvanil has been shown to inhibit transport of the endocannabinoid anandamide into RBL-2H3 cells and to inhibit the hydrolysis of anandamide by FAAH, the principal endocannabinoid metabolizing enzyme [29, 31]. The inability of rimonabant to attenuate the analgesic effects of olvanil suggests that activation of the endocannabinoid system, which has a well-documented inhibitory effect on sensory nerve function and TRPV1 activity, is unlikely to account for the differences between the pungency of olvanil and capsaicin, and, to the best of our knowledge, this is the first report evaluating the contribution of CB_1_ receptors to the effects of olvanil in vivo. In a recent study evaluating analgesic effects of olvanil on migraine, olvanil reduced spontaneous and stimulus-induced activity within the trigeminocervical complex; whereas it had no effect on cortical spreading depression; an effect mediated by both TRPV1 and CB_1_ [32].

To further explore the mechanisms that could mediate the anti-hyperalgesic effects of olvanil, we studied its effects on the capsaicin-evoked calcium responses in isolated DRG neurons. Despite both olvanil and capsaicin producing a robust increase in [Ca^2+^]_i_ in cultured dorsal root ganglion cells, olvanil was more effective than capsaicin at producing a desensitisation of TRPV1 to further capsaicin exposure. This finding along with a similar finding that the non-pungent capsaicin analogue palvanil produced a greater desensitising effect of TRPV1 compared to capsaicin [33], suggests that the desensitisation of the TRPV1 channel is not only dependant on the amount of agonist-stimulated calcium influx [34, 35], but also on the type of agonist applied. In line with this proposal, Xu and co-workers (2005) have shown that camphor, the active ingredient of many traditional balms and liniments, desensitizes TRPV1 in a calcium-independent fashion by activating TRPV1 via channel regions distinct from those affected by capsaicin [36]. Differences in the lipophilicity of the non-pungent TRPV1 agonists were previously suggested to explain the variation between them in vivo, in which highly lipophilic compounds were proposed to cause only a small Ca^2+^ influx via TRPV1 expressed in the plasma membrane, but not to be able to activate TRPV1 in the endoplasmic reticulum, in contrast to capsaicin (less lipophilic) which can activate both [37]. This observation is unlikely to explain the differences between olvanil and capsaicin responses in the present study as both induced similar calcium responses in DRG cells. This conclusion is supported by the finding that piperine, a pungent TRPV1 agonist causes a more marked desensitisation of TRPV1 compared to capsaicin even in a calcium-free medium [38]. Both piperine and capsaicin produce calcium influx in DRG cells [39], yet they do not cause the same neuroinflammatory peptides to be released. Capsaicin induces the release of substance P, serotonin and cholecystokinin, whereas piperine induces the release of substance P but not serotonin or cholecystokinin [40]. These differences in neurotransmitter release by different TRPV1 agonists may contribute to the differences in effects of olvanil and capsaicin in vivo. This concept is supported by a previous study showing that capsaicin induces a marked release of calcitonin gene related peptide (CGRP) while olvanil produces no detectable release of CGRP in rat spinal cord [41]. In agreement with the anti-hyperalgesic effects of olvanil observed in the present study, olvanil significantly reduced the release of CGRP evoked by depolarizing K^+^ stimulus [41]. These studies and the present findings clearly question the correlation between the calcium signals evoked by the TRPV1 agonists and their subsequent physiological effects.

In spite of the structural similarity between olvanil and capsaicin which suggests a similar mechanism of action, multiple agonist binding sites for these agonists could explain the differences observed between their responses in vivo, especially when considering the fact that TRPV1 is likely to present as a tetrameric complex [42]; therefore, different agonists could recruit more TRPV1 subunits or have alternative binding sites on the TRPV1 channel complex.

One limitation of our calcium imaging experiments is that they were performed on the cell bodies of primary afferent neurons (as in the majority of the previous studies using DRG) as axons are removed in the isolation process, raising the possibility that the TRPV1 channels expressed in the cell body have different properties from those expressed in the nerve terminals, as was recently proposed [43]. In this previous study, pungent compounds such as capsaicin and piperine amplified the glutamatergic spontaneous excitatory synaptic transmission in spinal substantia gelatinosa neurons, while olvanil, even at a very high concentration (10 μM), had a minimal effect. Thus, it is possible that pungent and non-pungent agonists could have similar effects on the cell body TRPV1 channels but might (in the case of pungent agonists) or might not (in the case of non-pungent agents) be able to generate action potentials, and subsequently nociceptive signals, when applied to TRPV1 expressed on the sensory nerve terminals. This possibility was recently studied in vagal C- fibres, in which the authors showed that jugular C-fibres have different capacities to transport particular TRPV1 agonists across cell membranes in the nerve terminals to intracellular binding sites, while the effects of different agonists on sensory nerve cell bodies were the same [44]. This undoubtedly needs to be verified in more integrated neuronal preparations in which measurements of [Ca^2+^]_i_ and applications of different agonists take place in the neuronal processes rather than in isolated DRG cell bodies.

## Conclusions

Olvanil was effective at reducing capsaicin-induced thermal hyperalgesia, likely via directly desensitizing TRPV1. This effect was independent of CB_1_ receptor activation. Although our findings cannot fully explain the lack of pungency of olvanil, they clearly support the possibility of employing olvanil to develop new topical treatments for a wide range of chronic pain conditions, such as arthritis, while overcoming the unwanted side effects of the currently used capsaicin preparations.

## Abbreviations

[Ca^2+^]_i_, intracellular calcium concentrations; CB_1_, cannabinoid receptor 1; DRG, dorsal root ganglion; TRPV1, transient receptor potential vanilloid type-1
